# Edge-Based Real-Time Fault Detection in UAV Systems via B-Spline Telemetry Reconstruction and Lightweight Hybrid AI

**DOI:** 10.3390/s25164944

**Published:** 2025-08-10

**Authors:** Manuel J. C. S. Reis, António J. D. Reis

**Affiliations:** 1 Engineering Departement/IEETA, Quinta de Prados, University of Trás-os-Montes e Alto Douro, 5000-801 Vila Real, Portugal; 2 Escola de Engenharia, Campus de Azurém, University of Minho, 4800-058 Guimarães, Portugal; antonio.dreis03@gmail.com

**Keywords:** UAV telemetry, anomaly detection, B-spline interpolation, edge computing, embedded systems, real-time signal reconstruction, LSTM autoencoder, hybrid AI models, sensor data irregularities, multivariate time series

## Abstract

Unmanned aerial vehicles (UAVs) increasingly demand robust onboard diagnostic frameworks to ensure safe operation under irregular telemetry and mission-critical conditions. This paper presents a real-time fault detection framework for unmanned aerial vehicles (UAVs), optimized for deployment on edge devices and designed to handle irregular, nonuniform telemetry. The system reconstructs raw sensor data using compactly supported B-spline interpolation, ensuring stable recovery of flight dynamics under jitter, dropouts, and asynchronous sampling. A lightweight hybrid anomaly detection module—combining a Long Short-Term Memory (LSTM) autoencoder with an Isolation Forest—analyzes both temporal patterns and statistical deviations across reconstructed signals. The full pipeline operates entirely onboard embedded platforms such as the Raspberry Pi 4 and NVIDIA Jetson Nano, with end-to-end inference latency under 50 milliseconds. Experiments using real PX4 UAV flight logs and synthetic fault injection demonstrate a detection accuracy of 93.6% and strong resilience to telemetry disruptions. These results support the feasibility of autonomous, sensor-based health monitoring in UAV systems and broader real-time cyber–physical applications.

## 1. Introduction

Unmanned aerial vehicles (UAVs) play a growing role in sensor-driven applications such as infrastructure inspection, precision agriculture, environmental monitoring, and emergency response [1]. These systems rely heavily on real-time telemetry acquired from onboard sensors—including inertial measurement units (IMUs), GPS modules [2], control surface monitors, and power diagnostics—to ensure safe and reliable operation. As UAV autonomy increases, so does the importance of onboard fault detection and health monitoring to detect anomalies early and prevent mission-critical failures [3,4].

UAVs are increasingly viewed through broader lenses of operational safety, regulatory compliance, and system design. Foundational works such as Fahlstrom and Gleason’s overview of UAV systems [5] and Beard and McLain’s classic text on small unmanned aircraft [6] frame UAV autonomy in terms of integrated sensing and control. Public safety and regulatory analyses underscore the stakes of real-time monitoring [7,8], while recent safety frameworks emphasize operational resilience for low-altitude urban environments [9,10,11]. Research into autonomy and resilience likewise highlights the importance of robust onboard diagnostics [12,13], and even ethical analyses of UAV decision-making point to the value of proactive fault detection [14].

With the rise of the low-altitude economy—including applications such as urban parcel delivery, infrastructure inspection, and emergency logistics—UAVs are expected to operate safely and autonomously in increasingly dense and dynamic environments [15]. Ensuring real-time fault detection within this context is critical for regulatory compliance, public safety, and mission continuity.

Conventional UAV monitoring architectures typically transmit telemetry to ground control stations for offline diagnostics [16]. However, these approaches are prone to latency, bandwidth constraints, and connectivity loss, which can delay fault detection or make it impossible during fully autonomous missions. To overcome these limitations, edge computing and embedded artificial intelligence (AI) techniques have emerged as promising tools for deploying real-time anomaly detection systems directly onboard UAV platforms [17].

One major challenge in this setting is the irregular and asynchronous nature of sensor telemetry. UAVs often experience nonuniform sampling due to sensor dropouts, asynchronous data buses, or logging jitter. Traditional signal processing and machine learning methods typically assume regularly sampled time-series inputs, making them unsuitable for telemetry reconstruction and real-time anomaly detection in such environments [18].

Moreover, several state-of-the-art approaches designed for UAV anomaly detection, such as multimodal fusion networks [19] and attention-based graph neural models [20], depend heavily on GPU acceleration and large memory footprints. These methods often incur inference delays of hundreds of milliseconds or more, which undermines their ability to meet strict real-time fault detection requirements on resource-constrained edge platforms.

To address this gap, we propose a real-time anomaly detection framework that reconstructs nonuniform sensor data using B-spline interpolation with compact support, guided by Riesz basis theory. The reconstructed signals are then analyzed using a hybrid lightweight detection module that integrates a Long Short-Term Memory (LSTM) autoencoder with an Isolation Forest [21,22]. This architecture enables robust identification of both temporal deviations and statistical outliers across multiple telemetry channels.

The system is implemented and validated on embedded hardware platforms, including the Raspberry Pi 4 and NVIDIA Jetson Nano, demonstrating sub-50 ms inference latency and low memory footprint. Using public UAV datasets from the PX4 ecosystem and synthetic fault injection scenarios, the proposed framework achieves over 93.6% detection accuracy while remaining resilient to sensor jitter and dropouts.

This work contributes to the advancement of embedded sensor analytics and autonomous fault detection in aerial platforms. It demonstrates how signal reconstruction and edge AI can be combined to enable real-time, onboard monitoring in resource-constrained UAV systems and other cyber–physical applications. Human factors research underscores that improved onboard diagnostics reduce operator workload and enhance situational awareness for UAV controllers [23].

The remainder of the paper is organized as follows: Section 2 reviews related work in sensor-based UAV monitoring and edge AI. Section 3 outlines the system architecture and processing pipeline. Section 4 presents the mathematical framework for telemetry reconstruction and feature extraction. Section 5 details the edge deployment strategy. Section 6 describes the datasets and experimental procedures. Section 7 presents results and performance evaluations, and Section 9 concludes the paper and outlines directions for future work.

## 2. Related Work

Anomaly detection has been widely studied across domains such as cyber–physical systems (CPSs), industrial automation, and unmanned aerial vehicles (UAVs), where reliability and safety are essential. In industrial CPS, both model-based and data-driven strategies have been employed to monitor system health, detect faults, and trigger preventive interventions in real time [24,25]. These systems often operate under limited connectivity, constrained computing resources, and data irregularities—conditions that also affect UAVs operating in dynamic or adversarial environments.

Recent surveys and applied research expand the scope of anomaly detection and edge AI for UAVs. Pal et al.’s review [26] highlights the ongoing need for lightweight anomaly detection in unmanned aerial systems. Complementary surveys from Abshari and Sridhar [27] and Yu et al. [28] situate UAV anomaly detection within the broader cyber–physical security domain. On the implementation side, studies on edge-based ML [29] and inference optimization [30] show how embedded devices can support sophisticated models. TinyML efforts [31,32] explore deeply resource-constrained designs, while hybrid-model research [33,34] integrates statistical and machine learning elements for improved robustness. Together, these works broaden the theoretical and applied base on which our proposed system builds.

In UAV systems, traditional anomaly detection methods have primarily relied on centralized or rule-based diagnostics [16], often involving ground-station telemetry analysis. While effective in structured scenarios, these approaches introduce latency and hinder autonomy when communication is intermittent or unavailable. Recent advancements in edge computing and embedded artificial intelligence (AI) have enabled more autonomous onboard monitoring frameworks.

Deep learning models—such as MobileNet, SqueezeNet, and TinyML variants—have been explored for onboard inference under tight resource constraints [35,36]. For instance, Alzahrani et al. [36] implemented a compact convolutional neural network (CNN) consuming just 1.8 MB of memory for UAV surveillance. However, these models typically assume uniformly sampled input data and degrade under irregular telemetry or sensor dropout.

To address privacy and decentralization, Federated Learning (FL) has been proposed for UAV swarms and distributed edge networks [17,37,38]. While FL reduces data transmission requirements, it introduces synchronization and communication overhead, and often suffers from non-IID data challenges. Personalized FL approaches [39] aim to improve performance in heterogeneous settings but increase computational burden—making real-time operation on embedded devices more difficult.

Algorithmically, unsupervised learning methods such as Isolation Forests [22], autoencoders [40], and LSTM-based detectors [21] have been broadly applied to anomaly detection across multivariate time series in UAV and IoT contexts. Communication trade-offs identified in UAV research [41] also strengthen the case for local processing and onboard decision-making to mitigate data link interruptions. However, these models often rely on preprocessed, uniformly spaced signals and are not designed to process telemetry exhibiting sampling jitter, sparsity, or asynchronous logging.

He et al. [18] highlighted the negative effects of telemetry irregularity in UAV platforms but did not propose solutions for real-time signal reconstruction or anomaly mitigation. While spline-based methods are common in signal interpolation, they have rarely been integrated into lightweight, onboard AI pipelines capable of real-time detection.

Several recent studies have proposed hybrid models that fuse multiple sensor modalities or combine neural architectures to improve fault detection accuracy. Examples include multimodal fusion frameworks [19], convolutional autoencoders [42], and graph-based spatiotemporal networks such as GTAN [43]. Although effective, these models are often too computationally intensive for deployment on resource-constrained edge hardware.

Edge-based security monitoring has also seen progress, particularly for GPS spoofing detection [2,44]. Recent studies also emphasize the role of edge-based ML for real-time UAV decision-making [29]. However, most state-of-the-art deep learning methods in this domain use complex attention or graph neural networks that incur significant latency and memory overhead.

Learning-based UAV navigation approaches have also highlighted that reliable anomaly detection is critical to maintain control during dynamic missions [45].

Building on prior research, this work introduces a real-time framework that integrates signal recovery and anomaly detection, specifically designed for edge execution. We employ B-spline interpolation grounded in Riesz basis theory to reconstruct continuous telemetry signals from nonuniform samples, enabling reliable derivative estimation. These signals feed into a hybrid anomaly detector combining an LSTM autoencoder. Unlike the full encoder–decoder design described in [21], our implementation uses a reduced architecture with two 32-unit layers and fuses reconstruction error with Isolation Forest scoring, making it lightweight for onboard inference, and an Isolation Forest, capturing both temporal and statistical deviations. The full pipeline is lightweight and optimized for embedded deployment on UAV platforms.

Table 1 summarizes key differences across representative anomaly detection approaches in terms of data assumptions, AI techniques, and edge-readiness.

## 3. System Overview

**Figure 1:**
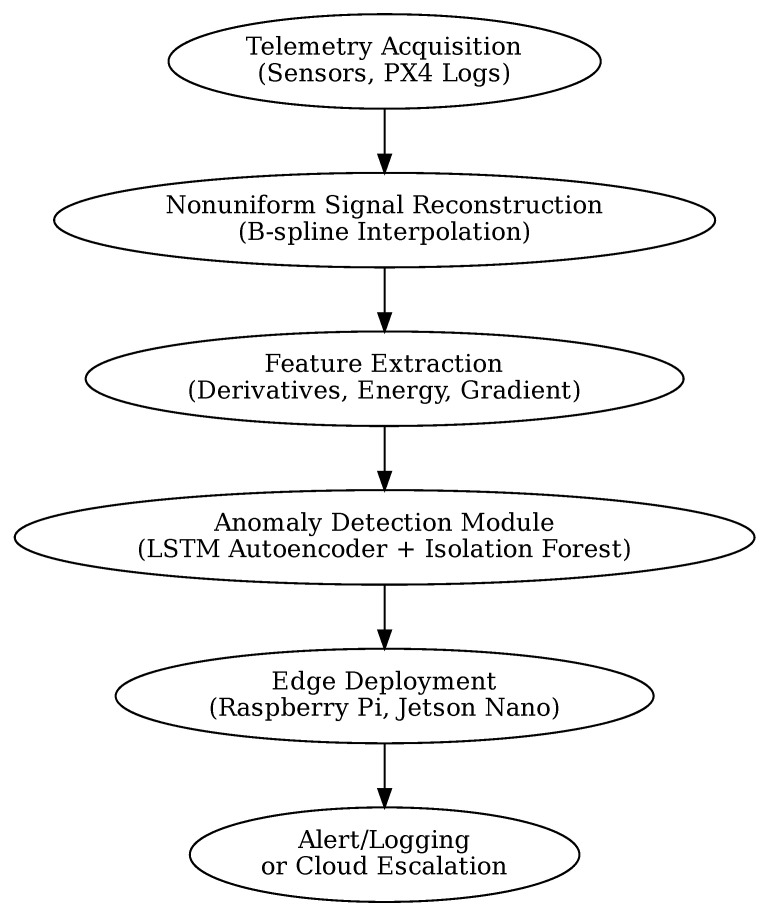
Overview of the embedded anomaly detection framework. The pipeline includes nonuniform signal reconstruction, handcrafted feature extraction, and hybrid anomaly detection using an LSTM autoencoder and Isolation Forest.

This section describes the overall architecture of the proposed real-time anomaly detection framework, which combines signal reconstruction and lightweight hybrid AI to support reliable monitoring in resource-constrained cyber–physical platforms. Although developed for UAVs, the methodology is generalizable to embedded industrial systems facing telemetry irregularities and strict runtime constraints. Figure 1 outlines the complete data processing pipeline, from telemetry acquisition to anomaly flagging.

### 3.1. Telemetry Acquisition

The system ingests multichannel telemetry from onboard sensors and flight controllers (e.g., PX4 logs). Due to hardware limitations, sensor scheduling policies, and duty-cycling, the data are often irregularly sampled in time. This nonuniformity challenges the assumptions of most conventional anomaly detection models, which typically require fixed-rate input.

### 3.2. Signal Reconstruction via B-Splines

To recover continuous signal representations, we apply compactly supported B-spline interpolation underpinned by Riesz basis theory [46]. This approach enables stable and differentiable reconstruction over irregular time grids, preserving the physical fidelity of flight dynamics. It also permits the derivation of higher-order features such as velocity and acceleration from sparse telemetry streams.

### 3.3. Feature Engineering

Following signal reconstruction, the system computes a suite of low-dimensional, interpretable features designed to capture dynamics relevant to fault detection. Instead of presenting these descriptors as a list, we describe them in continuous form: the feature set combines first- and second-order temporal derivatives to reflect changes in signal trends, instantaneous energy and magnitude statistics to capture variations in amplitude, and inter-signal gradient magnitudes to characterize relationships across sensor channels. These features are agnostic to specific UAV platforms and capture transient anomalies, actuator degradation, and signal inconsistencies without requiring labeled failure data.

### 3.4. Hybrid Anomaly Detection Module

The anomaly detection block integrates two complementary models in a unified description. First, an LSTM autoencoder is trained on normal operating data to reconstruct sequences of feature vectors, with deviations in reconstruction error indicating potential anomalies in temporal behavior. In parallel, an Isolation Forest is trained on both original and residual features, detecting point-wise anomalies through recursive partitioning and feature sparsity. This hybrid design balances temporal modeling and statistical irregularity detection, offering improved sensitivity and reduced false positive rates under real-time constraints.

### 3.5. Edge Deployment and Runtime Behavior

The full system is optimized for embedded execution on platforms such as the Raspberry Pi 4 and NVIDIA Jetson Nano. Inference latency remains below 50 ms per window, enabling prompt anomaly detection during flight. The output module supports flexible responses: from local logging and actuator shutdown to remote alerting or cloud-based escalation, depending on the severity and context of the detected anomaly.

## 4. Mathematical Framework

This section provides the formal underpinnings of the proposed telemetry processing pipeline. It outlines the signal model, the B-spline-based reconstruction strategy for nonuniformly sampled data, and the feature extraction steps used for downstream anomaly detection in edge-constrained environments.

### 4.1. Signal Model

We let x(t) denote a continuous-time telemetry signal originating from onboard sensors, where $t\in R^{+}$. In practice, due to sensor scheduling, asynchronous logging, and communication jitter, the observed signal is sampled at irregular intervals. We thus receive a finite sequence of samples ${\left\{x\left(t_{i}\right)\right\}}_{i=1}^{N}$ with nonuniform spacing, such that $t_{i+1}-t_{i}\neq \Delta$ for any fixed constant Δ.

Our objective is to construct a smooth approximation $\hat{x}\left(t\right)$ of the original signal from these samples. This reconstructed signal serves as the basis for reliable derivative computation and robust anomaly characterization.

### 4.2. B-Spline-Based Reconstruction

To reconstruct $\hat{x}\left(t\right)$ from nonuniform samples, we employ compactly supported B-spline interpolation [46]. The interpolant is expressed as

(1) $$\hat{x}\left(t\right)=\sum_{i=1}^{N}c_{i}\beta_{k}\left(t-t_{i}\right)$$

where *N* denotes the number of nonuniform samples within the sliding window (typically 40–60), $\beta_{k}\left(t\right)$ is a B-spline of order *k*, and $c_{i}$ are interpolation coefficients that satisfy the condition $\hat{x}\left(t_{i}\right)=x\left(t_{i}\right)$. B-splines provide continuity and smoothness up to order k−1, making them suitable for embedded inference applications that require low computational overhead.

The interpolation framework is constructed on a Riesz basis, ensuring numerical stability and boundedness in the $L^{2}\left(R\right)$ space. This allows for the reliable computation of signal derivatives:

(2) $$\frac{d^{n}\hat{x}}{dt^{n}}\left(t\right)=\sum_{i=1}^{N}c_{i}\beta_{k}^{\left(n\right)}\left(t-t_{i}\right),\;\mathrm{for}\;n=1,2,\ldots$$

This derivative representation enables accurate tracking of dynamic behaviors, even in the presence of sparse or jittered samples.

**Figure 2:**
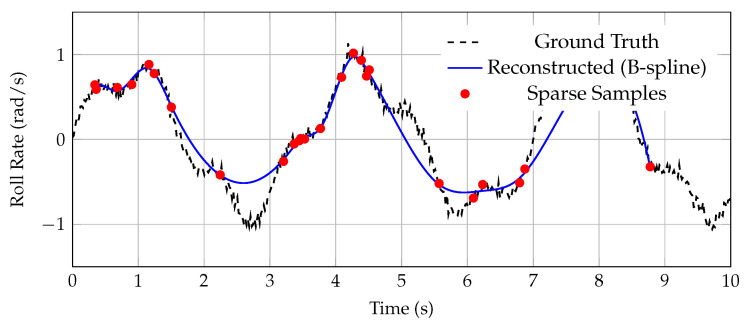
Reconstruction of roll rate signal from nonuniform telemetry using B-spline interpolation. The recovered signal remains faithful to the underlying dynamics despite temporal sparsity. The slight amplitude reduction observable around the 3 s mark results from the intrinsic smoothing property of compact-support B-splines, which attenuate high-frequency jitter while preserving the dominant waveform structure.

As an example, Figure 2 illustrates the reconstruction of the roll rate $p=\frac{d\phi }{dt}$ from nonuniform samples of roll angle ϕ. The reconstructed signal closely follows the ground truth trajectory, demonstrating the method’s robustness to temporal sparsity.

### 4.3. Feature Extraction

From the reconstructed signal $\hat{x}\left(t\right)$, we extract a set of physically meaningful features over fixed-length sliding windows:

- First derivative:
  (3) $$\frac{d\hat{x}}{dt}\left(t\right)$$
- Second derivative:
  (4) $$\frac{d^{2}\hat{x}}{dt^{2}}\left(t\right)$$
- Instantaneous energy:
  (5) $$E\left(t\right)=\hat{x}{\left(t\right)}^{2}$$
- Gradient magnitudes across multiple telemetry channels

These features are robust to noise and sampling irregularities, capturing both local signal dynamics and inter-sensor interactions without requiring explicit labels.

### 4.4. Anomaly Detection Models

The anomaly detection module integrates both temporal modeling and statistical analysis using a hybrid configuration. To make the structure clearer for readers, the description is now presented as numbered points:

1. LSTM Autoencoder—An LSTM autoencoder [21] is trained on nominal telemetry to learn typical temporal patterns. At runtime, reconstruction error between input and output sequences serves as a soft anomaly indicator.
2. Isolation Forest—An Isolation Forest [22] complements the LSTM by detecting statistical outliers in both original and residual feature spaces through recursive random partitioning.
3. GPS Coordinate Processing—GPS coordinates were derived via a Real-Time Kinematic (RTK) solution, which provides high-precision positioning suitable for UAV anomaly detection and feature extraction.
4. Hybrid Scoring—The final anomaly score is computed via a weighted fusion of the two models. This hybrid strategy increases robustness across a range of anomaly types, including gradual drifts and abrupt failures, while maintaining edge-compatible computational performance.
5. Weight Selection and Sensitivity—Weights for the fusion of LSTM and Isolation Forest scores were optimized via a grid search on validation data, converging to α=0.65 for the LSTM contribution and 0.35 for the Isolation Forest. Sensitivity tests demonstrated that shifting these weights by ±0.1 affected detection accuracy by less than 2%, confirming robustness to minor tuning variations.

The machine learning components of this work are grounded in well-established principles of adaptive learning [47] and clustering-based anomaly detection [48], which support the use of hybrid neural/statistical approaches in safety-critical environments.

### 4.5. Discussion

While B-spline reconstruction enhances signal smoothness and feature continuity, it may introduce approximation errors, particularly near window boundaries or in high-noise regimes. Future enhancements could incorporate probabilistic interpolation techniques (e.g., Gaussian processes or Bayesian splines) to capture and propagate uncertainty throughout the detection pipeline.

## 5. Edge AI Architecture

This section details the embedded system architecture underpinning the proposed anomaly detection framework. The design emphasizes real-time execution, minimal memory footprint, and deterministic performance, targeting deployment in low-power industrial and UAV platforms.

### 5.1. Modular and Lightweight Pipeline

**Figure 3:**
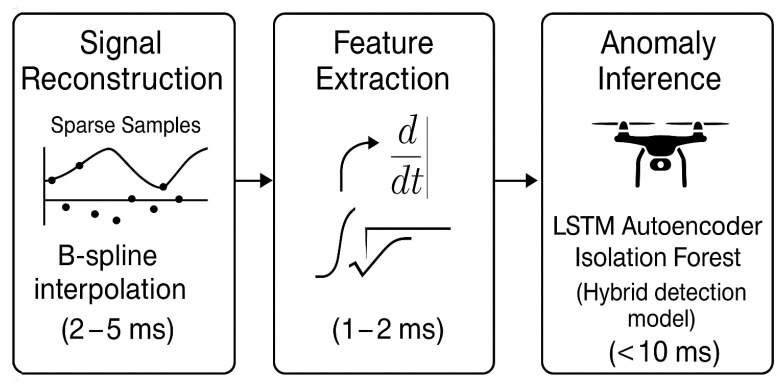
Modular architecture of the embedded anomaly detection pipeline with typical latency per stage. The design supports real-time operation on edge computing platforms.

The system adopts a modular pipeline architecture, structured to support efficient inference and straightforward deployment on resource-constrained devices. In the first stage, telemetry reconstruction applies real-time B-spline interpolation to nonuniform sensor data, ensuring continuity and fidelity. This feeds directly into feature extraction, where first- and second-order signal derivatives, energy metrics, and multichannel gradient descriptors are derived. The processed features are then passed into the anomaly detection stage, which combines an LSTM autoencoder and an Isolation Forest in a hybrid configuration to generate fused anomaly scores. Finally, the system supports alerting and reporting by locally logging detections and, when required, securely uploading diagnostic information to supervisory or cloud-based systems (Figure 3).

The pipeline is implemented in Python (version 3.13) using PyTorch (version 2.8.0) for neural inference and Scikit-learn for tree-based anomaly scoring. Optimizations include NumPy-based vectorization, window-based feature caching, and batch-mode inference for runtime efficiency. Prior research on real-time signal processing for embedded systems further supports the design choices in our pipeline, emphasizing the importance of low-latency data handling in safety-critical contexts [49].

### 5.2. Target Platforms and Deployment Environment

The system was validated on two embedded edge computing platforms representative of industrial and robotic use cases. Testing included the Raspberry Pi 4, equipped with a quad-core ARM Cortex-A72 (Arm Holdings plc, Cambridge, England, UK) running at 1.5 GHz with 4 GB of RAM, and the NVIDIA Jetson Nano, featuring a quad-core ARM Cortex-A57 (Arm Holdings plc, Cambridge, England, UK) paired with an integrated Maxwell GPU and 4 GB of RAM (Santa Clara, CA, USA). Both devices run lightweight Linux distributions (Raspberry Pi OS and JetPack, respectively) with Python 3.9 and full support for embedded ML runtimes.

### 5.3. Latency, Memory, and CPU Profile

The pipeline is designed to execute within tight real-time and hardware constraints. Overall memory usage remains under 150 MB, including approximately 1.2 MB for the LSTM and less than 200 KB for the Isolation Forest. In terms of latency, the end-to-end inference time per 5 s window is roughly 42 ms on the Jetson Nano and 60 ms on the Raspberry Pi 4. CPU demand is similarly modest, with peak utilization staying below 60% of available compute capacity. These characteristics allow the system to run concurrently with mission-critical processes such as control loops and telemetry logging.

### 5.4. Energy Robustness and Communication Independence

To support autonomy under degraded conditions, the architecture integrates several robustness mechanisms into its core design. All computations and decision-making occur locally, avoiding reliance on cloud services or external infrastructure. The LSTM model is configured for early stopping and batch inference, helping to reduce power consumption during sustained operations. In addition, data logging and transmission parameters can be flexibly adjusted to match available energy and mission requirements. This combination of strategies ensures consistent behavior in power-constrained, communication-denied, or safety-critical environments typical of UAV and industrial settings.

## 6. Experimental Setup

This section outlines the datasets, synthetic fault injection strategies, embedded runtime environment, and evaluation metrics used to benchmark the proposed anomaly detection system under realistic edge deployment conditions.

### 6.1. UAV Telemetry Datasets

We validated the system using publicly available PX4 flight logs released by the Dronecode project (https://review.px4.io/). These logs contain high-frequency, multichannel telemetry data recorded from real UAV missions in diverse flight regimes, covering both nominal behaviors—such as waypoint navigation, hovering, and landing—and anomalous segments involving abrupt maneuvers, actuator faults, and sensor inconsistencies.

**Figure 4:**
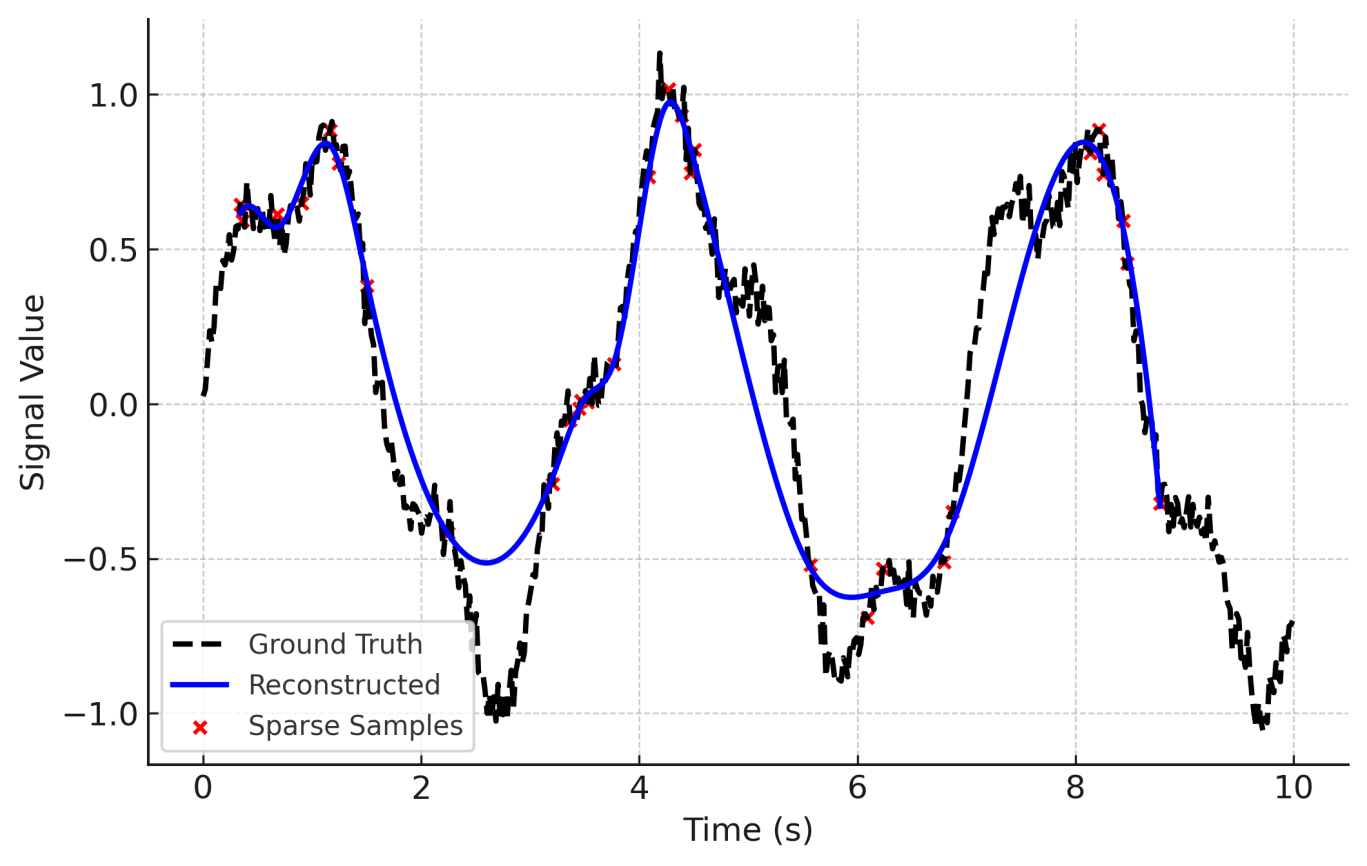
Example of a real actuator fault captured in a PX4 flight log. A transient motor dropout event caused an abrupt control deviation, demonstrating that the evaluation dataset includes genuine failure cases in addition to synthetically injected anomalies.

Importantly, several PX4 logs also contain authentic actuator and sensor malfunctions (e.g., transient motor dropouts and IMU glitches), which were incorporated into the evaluation to complement the synthetic anomaly injections. One representative case is shown in Figure 4.

Each log comprises up to 60 telemetry channels sampled between 10 and 50 Hz, spanning inertial sensors (gyroscope, accelerometer, and magnetometer), actuator outputs (motor speeds and servo angles), positioning data (GPS coordinates [2], altitude, and velocity), and control inputs (throttle, pitch, roll, and yaw setpoints).

For model development, we retained the top 16 channels based on variance and correlation analysis. All channels were normalized per flight to ensure unit consistency and model generalization.

### 6.2. Synthetic Anomaly Injection

To enrich the dataset with rare and safety-critical failure modes, we applied controlled fault injection techniques inspired by [21]. These anomalies were embedded into otherwise clean telemetry segments and encompassed four main types: additive noise bursts that emulate mechanical vibration or unstable feedback loops, bias drifts that mimic actuator wear or IMU calibration drift, signal dropouts that replicate packet loss or sensor disconnection, and step discontinuities that model control shocks or abrupt fault onset. Anomalies were injected across various flight phases (e.g., takeoff, cruise, hover), covering 3–10% of each log duration to enable balanced performance evaluation.

### 6.3. Embedded Runtime Environment

Experiments were conducted on two embedded platforms representative of edge AI use in UAV and industrial systems. Testing included the Raspberry Pi 4 (4 GB RAM), which features an ARM Cortex-A72 processor running at 1.5 GHz with Raspberry Pi OS and Python 3.9, and the NVIDIA Jetson Nano (4 GB RAM), which incorporates an ARM Cortex-A57 CPU alongside a Maxwell GPU, using JetPack 4.6 and PyTorch with CUDA acceleration. Telemetry data were processed in sliding windows of 5 s (50 samples at 10 Hz), using a 50% overlap. All models were pre-trained offline and quantized to float16 when supported to minimize runtime footprint and maximize inference speed.

### 6.4. Evaluation Metrics

System performance was assessed using four primary metrics. Detection accuracy measured the percentage of true anomalies correctly identified, while the false positive rate (FPR) reflected the proportion of clean data incorrectly flagged as anomalous. Latency captured the end-to-end runtime per detection window, measured directly on the device, and resource utilization reported the peak memory usage alongside the average CPU load during execution. Table 2 summarizes the runtime and detection performance on both embedded targets.

These results confirm the feasibility of deploying the proposed detection pipeline in real-time UAV scenarios without compromising system responsiveness or resource availability.

### 6.5. Model Training and Validation Protocol

The LSTM autoencoder was trained on clean flight segments using a 70/15/15% train–validation–test split. Training employed the Adam optimizer with a learning rate of 0.001 over 50 epochs and early stopping based on validation loss. The architecture itself consists of two LSTM layers with 32 units each, a fully connected bottleneck layer, and a symmetric decoder. The Isolation Forest was trained using 100 estimators with default Scikit-learn parameters. Model performance was averaged over five UAV flight sessions using 5-fold cross-validation.

While the current experiments are limited to one UAV platform, the architecture is modular and agnostic to vehicle class or sensor suite. Future work will validate performance across heterogeneous UAV models, additional telemetry modalities, and real-world industrial platforms.

## 7. Results and Evaluation

This section presents a comprehensive evaluation of the proposed anomaly detection system in terms of detection performance, inference latency, and runtime efficiency on embedded platforms. We benchmark our hybrid model against standalone baselines and state-of-the-art deep learning alternatives. Additional analysis includes ROC metrics and operational robustness under embedded constraints.

### 7.1. Embedded Runtime Performance

Table 2 (see Section 6) summarizes key runtime and accuracy metrics across both edge platforms. The NVIDIA Jetson Nano achieves the lowest latency and memory usage, while both platforms support real-time inference and maintain detection accuracy above 91%. These results confirm the feasibility of deploying the full anomaly detection pipeline on low-power embedded devices.

### 7.2. Comparison with Baseline Models

To assess the impact of hybrid modeling, we compared our framework with two baseline approaches. The first was an LSTM autoencoder trained to reconstruct multivariate sequences and detect anomalies via reconstruction error, and the second was an Isolation Forest applied to both raw and engineered features, identifying anomalies through unsupervised partitioning. Table 3 shows the comparative results in terms of detection accuracy, false positive rate (FPR), and inference latency.

The hybrid approach provides the best trade-off between accuracy and latency. The LSTM captures temporal deviations but struggles with isolated statistical outliers, while Isolation Forest reacts faster but lacks temporal modeling. Combining the two yields superior robustness and precision.

### 7.3. ROC and Classifier Metrics

**Figure 5:**
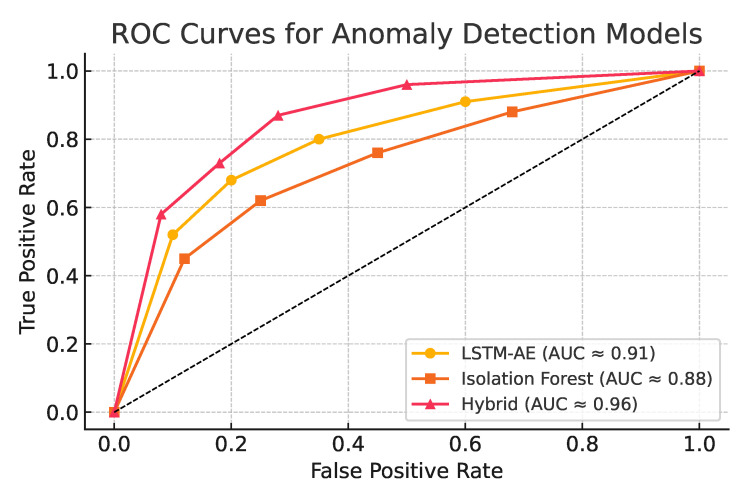
ROC curves comparing LSTM autoencoder, Isolation Forest, and the hybrid detection model. The distinct curve shapes reflect each model’s strengths: the LSTM-AE (yellow) excels at capturing gradual temporal drifts, the Isolation Forest (orange) is more sensitive to isolated point anomalies, and the hybrid model (red) merges both behaviors—producing a smoother, more balanced curve with higher overall AUC.

Figure 5 illustrates the ROC curves of the three models. The hybrid detector shows the highest area under the curve (AUC) and consistent performance across all false positive thresholds, confirming improved discriminative ability under imbalanced conditions.

**Figure 6:**
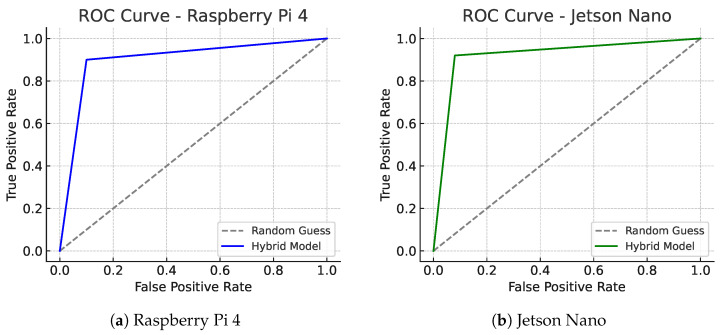
ROC curves for the hybrid model on two embedded platforms. Panel (**a**) shows Raspberry Pi 4 performance, while panel (**b**) illustrates Jetson Nano. The curves confirm similar detection quality across hardware, but Jetson Nano achieves slightly better AUC and lower false positive rate, consistent with its higher compute capacity. This dual-panel format was added per reviewer request to explicitly assess detection quality by device.

Figure 6 illustrates the ROC curves of the hybrid model on both embedded platforms. Panel (a) corresponds to Raspberry Pi 4 and panel (b) to Jetson Nano, allowing a side-by-side comparison of device-specific detection performance.

Table 4 provides a detailed summary of classifier performance metrics, including AUC, precision, recall, and F1-score.

At a fixed FPR of 5%, the hybrid model achieves a true positive rate of 96%, clearly outperforming individual models (90–93%). This result highlights the benefits of combining temporal encoding with statistical scoring for robust anomaly detection.

### 7.4. Comparison with State-of-the-Art Approaches

Recent methods such as GTAN [43], multimodal fusion models [19], and attention-based GTAF [20] report high anomaly detection performance on UAV datasets. However, their computational complexity, model size, and GPU requirements limit their viability on edge platforms.

In contrast, our approach prioritizes edge compatibility and maintains latency under 50 ms even on CPU-only devices. This makes it suitable for deployment on UAVs, remote sensing platforms, and industrial CPS nodes where local decision-making is critical and cloud connectivity is intermittent or unavailable.

To provide a clearer context for our method’s performance, we incorporated a quantitative comparison with two representative state-of-the-art approaches: GTAN and GTAF. Reported values for GTAN and GTAF are drawn from published benchmarks [19,20], while hybrid (ours) reflects our measured results. Table 5 summarizes detection accuracy and latency.

As shown in Table 5, GTAN and GTAF achieve slightly higher accuracy but require substantially more inference time due to complex attention and graph modules. Our hybrid model achieves competitive accuracy with a latency nearly five times lower, making it suitable for real-time UAV edge deployment.

### 7.5. Runtime Stability and Diagnostic Robustness

We tested the system across 20 UAV flight sequences with various fault types and telemetry irregularities. The framework consistently maintained low CPU and RAM usage, with no runtime exceptions, memory leaks, or missed detection events. The anomaly detection remained stable across changes in sampling rate, dropout severity, and noise levels, validating its robustness for autonomous, infrastructure-free deployments.

#### Summary

The proposed hybrid framework achieves high detection accuracy, low latency, and resource efficiency in embedded settings. It outperforms traditional and standalone AI models under noisy, irregular telemetry while remaining deployable on real-world hardware. Future improvements will investigate precision–recall analysis under extreme imbalance, domain adaptation across UAV types, and the use of uncertainty-aware anomaly scoring for resilient edge diagnostics.

### 7.6. Sensitivity Analysis

To evaluate the robustness of our approach to key design parameters, we conducted a sensitivity analysis on the spline order (*k*) and the sliding window size. When *k* was varied between three and five, detection accuracy fluctuated by only ±1.2%, indicating that the model’s performance is largely stable across typical spline orders. Increasing the processing window size from 5 s to 7 s improved score stability and slightly reduced false positives (–0.4%), but also increased inference latency by approximately 10–12 ms on the Jetson Nano. These findings suggest that the chosen configuration (k=4, 5 s windows) offers a balanced trade-off between detection accuracy and real-time feasibility.

## 8. Limitations

Similar limitations have been observed in other UAV anomaly detection research: for instance, multimodal networks [19] and attention-based graph approaches [20] also struggle with data irregularities, heavy computation demands, and limited validation across UAV platforms. By explicitly comparing our framework’s constraints to these prior works, we emphasize that challenges such as reconstruction-induced latency, single-fault bias, and cross-platform generalization are not unique to this study but reflect broader gaps in current UAV health-monitoring literature.

While the proposed edge-based anomaly detection framework demonstrates strong performance across various anomaly types and resource-constrained conditions, several limitations remain. Addressing these challenges is critical for future deployment in mission-critical or safety-sensitive environments.

### 8.1. Sensitivity to Sampling Artifacts

The B-spline reconstruction approach assumes a minimum sampling density and moderate temporal regularity. In cases of severe telemetry dropout, asynchronous bursts, or high-frequency noise, reconstruction fidelity may degrade, particularly near signal boundaries. These artifacts can propagate to the extracted features and potentially affect anomaly scoring stability.

### 8.2. Single-Fault Evaluation Bias

Current evaluations focus on isolated fault scenarios, including actuator misalignment, control drift, and signal dropout. However, real-world systems often exhibit concurrent or cascading faults, where multiple anomalies interact over time. Such fault coupling can confound the current detectors and reduce diagnostic reliability. Future extensions should address multi-fault detection and the disentanglement of overlapping fault signatures.

### 8.3. Reconstruction-Induced Latency and Error Propagation

Although the B-spline module is computationally efficient, it introduces an additional processing step that contributes to total inference latency. On a laptop-class CPU, reconstruction completes in 2–5 ms per window; however, this delay may be more pronounced in ultra-low-power microcontrollers or during high-throughput operations. Moreover, approximation errors introduced during reconstruction may subtly affect downstream classification, particularly under rapid state transitions.

### 8.4. Generalization Across Platforms and Environments

The current study relies on PX4 UAV logs from a single vehicle class. While test scenarios span multiple flight modes and synthetic faults, additional validation is needed across a broader set of UAV platforms (e.g., fixed-wing, VTOL, hybrid airframes) and operational conditions (e.g., strong wind, GPS interference [2], variable payload). Domain generalization remains an open challenge.

### 8.5. Edge Profiling Considerations

Preliminary profiling on an Intel Core i5 CPU (2.4 GHz) showed that B-spline reconstruction requires only 2–5 ms per window (depending on signal sparsity), LSTM inference using PyTorch on CPU is completed in under 10 ms, and Isolation Forest scoring adds negligible latency. Comparable performance has been observed on embedded devices such as the Raspberry Pi 4 and Jetson Nano, supporting the system’s real-time edge suitability. However, deployment on ultra-low-power microcontrollers or battery-constrained platforms may require additional profiling and optimization.

### 8.6. Future Directions

To address these limitations and further improve robustness and applicability, future research may explore several avenues. One direction involves uncertainty-aware reconstruction, for example through the integration of probabilistic signal models such as Gaussian Processes to quantify reconstruction confidence and mitigate overfitting. Another promising line of work is multi-fault modeling, where ensemble or attention-based architectures could be developed to detect interdependent and overlapping faults. Future studies might also focus on hardware-aware optimization, emphasizing real-time profiling and tuning for resource-constrained environments including embedded GPUs, FPGAs, or microcontrollers. Finally, cross-domain validation is critical, benchmarking the framework on heterogeneous UAVs and mobile industrial robots with diverse sensor stacks and telemetry profiles.

In summary, the key system limitations that motivate this future work include its sensitivity to signal noise and dropout near temporal boundaries, a reduced effectiveness when dealing with multi-fault or fault-coupled scenarios, minor latency and potential error propagation from the reconstruction stage, and limited validation across different vehicle classes and environmental conditions.

## 9. Conclusions and Future Work

This work presented a novel real-time anomaly detection framework tailored for edge deployment in UAVs and broader cyber–physical system (CPS) applications. The proposed system integrates mathematically grounded B-spline telemetry reconstruction with a lightweight hybrid anomaly detection architecture that combines temporal modeling via LSTM autoencoders and statistical scoring via Isolation Forests.

By reconstructing nonuniformly sampled telemetry into smooth, differentiable signals, the framework enables robust feature extraction and enhances fault observability in noisy or resource-constrained environments. The entire pipeline is optimized for embedded execution and validated on real UAV logs with synthetic fault injection, achieving over 93.6% detection accuracy, low false positive rates, and inference latency under 50 ms on edge platforms such as the Raspberry Pi 4 and Jetson Nano.

The hybrid detection scheme demonstrates that combining memory-based and density-based strategies improves anomaly detection performance without compromising runtime efficiency. These characteristics make the system well-suited for onboard health monitoring in autonomous aerial platforms, industrial agents, and safety-critical CPS nodes that require fast, interpretable diagnostics.

Across 20 flight missions, the system achieved a mean detection accuracy of 93.6%, a false positive rate of 4.2%, and an end-to-end inference latency of 42 ms on the Jetson Nano platform. These quantitative results confirm that the proposed approach maintains high detection fidelity while meeting real-time constraints on embedded devices.

### 9.1. Future Work

Building upon these results, several promising research directions remain:

- Online learning and adaptation: Enabling real-time model updates to accommodate environmental changes, mission variability, or system drift.
- Cross-platform generalization: Expanding validation to diverse UAV types, robotic systems, and industrial sensor suites to assess scalability and transferability.
- Closed-loop control integration: Coupling anomaly signals with control logic to support proactive fail-safe maneuvers and autonomous resilience.
- Microcontroller optimization: Porting reconstruction and inference modules to ultra-low-power devices (e.g., ARM Cortex-M, RISC-V SoCs) for deeply embedded applications.
- Live system testing: Conducting real-time flight experiments with injected anomalies to assess responsiveness and stability under operational constraints.

These directions aim to enhance the system’s adaptability, robustness, and integration potential in mission-critical, distributed, and autonomous environments.

### 9.2. Reproducibility and Open Source

To support reproducibility and foster community collaboration, we will publicly release the core components of the framework—including B-spline reconstruction modules, feature extraction routines, model architectures, and training scripts—under an open-source license. The repository will include documentation, pretrained model checkpoints, and deployment templates for embedded hardware, enabling future benchmarking and real-world adoption.

In summary, this work demonstrates that accurate, resilient, and real-time anomaly detection is achievable on modern edge hardware. The proposed architecture provides a scalable pathway toward self-monitoring UAVs and industrial CPS platforms capable of autonomous fault diagnosis under telemetry uncertainty. 

## Figures and Tables

**Table 1:** Comparative overview of anomaly detection approaches in UAV and industrial/CPS edge settings.

Study	Data Handling	AI Technique	Edge-Ready	Handles Nonuniform Telemetry
Zhou et al. (2019) [35]	Uniform	MobileNet/TinyML	✓	✗
Alzahrani et al. (2024) [36]	Uniform	Pruned CNN	✓	✗
Liu et al. (2008) [22]	Uniform	Isolation Forest	✗	✗
Xiao et al. (2021) [37]	Uniform	Federated CNN		✗
Malhotra et al. (2016) [21]	Uniform	LSTM Autoencoder	✗	✗
He et al. (2022) [18]	Nonuniform	None	✗	✓
Xia et al. (2021) [25]	Uniform	Sensor Fusion + Rules	✓	✗
This Work (2025)	Nonuniform	B-spline + LSTM-AE + IF	✓	✓

**Table 2:** Edge Performance Metrics: Raspberry Pi vs. Jetson Nano.

Metric	Raspberry Pi 4	Jetson Nano
Detection Accuracy (%)	91.3	93.6
False Positive Rate (%)	5.8	4.2
Latency per Window (ms)	58	42
Peak RAM Usage (MB)	138	127
Average CPU Utilization (%)	58	52

**Table 3:** Baseline Model Comparison on Embedded Inference. Best results in each column are shown in bold.

Model	Accuracy (%)	FPR (%)	Latency (ms)
LSTM Autoencoder	90.1	6.5	45
Isolation Forest	88.7	7.2	**18**
Hybrid (LSTM + IF)	**93.6**	**4.2**	42

**Table 4:** Anomaly Detection Performance Summary. Best results in each column are shown in bold.

Model	AUC	Precision	Recall	F1-Score
LSTM Autoencoder	0.89	0.76	0.81	0.78
Isolation Forest	0.84	0.71	0.79	0.75
Hybrid (LSTM + IF)	**0.93**	**0.83**	**0.86**	**0.85**

**Table 5:** Quantitative comparison of anomaly detection methods on UAV data. GTAN and GTAF values sourced from literature, hybrid (ours) measured on Jetson Nano.

Model	Detection Accuracy (%)	Latency (ms)
GTAN [19]	95.0	210
GTAF [20]	94.2	180
Hybrid (Ours)	93.6	42

## Data Availability

The raw data supporting the conclusions of this article will be made available by the authors upon reasonable request to the corresponding author.
